# EHMTI-0394. Predictive parameters for the effect of botulinum toxin infiltrations in chronic migraine

**DOI:** 10.1186/1129-2377-15-S1-M1

**Published:** 2014-09-18

**Authors:** BA Bergmans, R Bruffaerts, M Verhalle, L Vanopdenbosch, K Verhoeven, A Van Dycke, O Deryck

**Affiliations:** 1 Neurology, AZ St-Jan Brugge-Oostende AV Campus Brugge, Brugge, Belgium; 2 Neurology Neurosciences, UZ Leuven Laboratory for Cognitive Neurology University of Leuven, Leuven, Belgium; 3 Neurology, AZ St-Jan Brugge-Oostende AV Campus Henri Serruys, Oostende, Belgium

## Introduction

Since the PREEMPT trials botulinum toxin infiltrations have become mainstay treatments for chronic migraine.

Although not reimbursed in Belgium, botulinum toxin infiltrations can be a viable option for refractory chronic migraine patients in whom previous oral treatments have failed.

## Aims

We report on the effectiveness and side effects of botulinum toxin infiltrations for chronic migraine.

We investigate which parameters influence the effect of the treatment.

## Methods

Patients meeting criteria for chronic migraine who had failed conventional oral treatments for the condition were proposed botulinum toxin infiltrations. After informed consent, they were asked to keep detailed headache calendars, documenting the number of headache days, the number of migraine days, the intensity of the headaches/migraines and analgetic use. We compared these parameters before treatment and after two cycles of botulinum toxin infiltrations. Statistical significance was calculated by means of a Wilcoxon signed rank test.

## Results

In our preliminary cohort of 7 patients treated with botulinum toxin no major adverse events were reported. One patient reported a mild transient unilateral ptosis that did not hamper vision.

On average we observed a reduction in headache days from 26.6 days/month before treatment to 22.0 days/month after treatment (p=0.250). The number of migraine days after 2 cycles was reduced on average from 15.6 to 8.2 migraine days/month (p=0.016). The average reduction of the number of days that painkillers were used after 2 treatment cycles, was much less pronounced, from 9.1 to 7.6 days/month (p=0.406).

## Conclusions

In our experience, even though high doses of botulinum toxin are used in chronic migraine, the treatment is safe.

In selected patients with refractory chronic migraine botulinum toxin infiltrations can significantly improve the headache control. We observed that there is a subpopulation of patients with a distinct and significant effect of the infiltrations who will continue treatment beyond 2 treatment cycles. Another subpopulation seems to have little or no benefit from the infiltrations and will discontinue treatment after 2 cycles.

We will investigate in a regression analysis in 20 patients which patient characteristics influence the success of the botulinum toxin treatments.

